# Histology and gametogenesis in *Heleobia piscium* (Cochliopidae) from the Multiple Use Reserve “Isla Martín García,” Buenos Aires, Argentina

**DOI:** 10.7717/peerj.2548

**Published:** 2016-10-06

**Authors:** Stella Maris Martin, Ana C. Díaz

**Affiliations:** 1 División Zoología Invertebrados-FCNyM-UNLP-Investigador Independiente CIC, Universidad Nacional de La Plata, Buenos Aires, Argentina; 2 División Zoología Invertebrados-FCNyM-UNLP-Becaria Doctoral CONICET, Universidad Nacional de La Plata, Buenos Aires, Argentina

**Keywords:** Gametogenesis, Histology, *Heleobia*, Isla Martín García, Argentina

## Abstract

*Heleobia piscium* (d’Orbigny, 1835), a member of the Cochliopidae family found only in South America, is distributed from Entre Ríos, Delta del Paraná, and the littoral of the Río de la Plata down as far as to Punta Indio (Buenos Aires), the southernmost limit of the snail’s geographical distribution. To date, little information is available regarding the reproductive cycle of species within this family either in Argentina or throughout South America. The present work analyzed the histology of the reproductive system of the gonochoric species *H. piscium* and determined the stages oogenesis and spermatogenesis under natural conditions. Specimens of *H. piscium* were collected in the Multiple-Use Natural Reserve Isla Martín García, located in the Upper Río de la Plata estuary to the south of the mouth of the Uruguay River. The gametogenic cycle in both sexes was found to consist of the following stages: early maturation, maturation, and evacuation. The maturation period was found to extend from January to October and evacuation of the gametes to start in November and end in February (summer in the Southern Hemisphere). The results indicated the *H. piscium* exhibit a reproductive cycle without a resting period.

## Introduction

*Heleobia* Stimpson, 1865 is a genus within the family Cochliopidae Tryon, 1866 (Wilke et al., 2001; Szarowska, Falniowski & Steffek, 2011) comprising 101 species, of which 90 are found in South America (Hershler & Thompson, 1992; Pons da Silva & Veitenheimer-Mendes, 2004; Cazzaniga, 2011; Collado, 2015). Rumi et al. (2006) and Rumi et al. (2008) reported 16 *Heleobia* species for Argentina, of which 10 are endemic.

*Heleobia piscium* (d’Orbigny, 1835), a South American species, is distributed from Entre Ríos, Delta del Paraná, and the littoral of the Río de la Plata estuary down to Punta Indio (Buenos Aires), the southernmost limit of the snail’s geographical range. Research on the Cochliopidae to date has tended to focus on the taxonomy, morphology, and biology of the family, but little information is available on certain aspects of the anatomy, life cycle, reproduction, dispersal strategies, behavior, and genetics of its species. Accordingly, few studies have dealt with the reproductive cycle species from Argentina or from South America, except for those conducted by Cazzaniga (1982) on *Littoridina parchappii* (d’ Orbigny, 1835) in the drainage channels of the lower valley of the Colorado-River province of Buenos Aires, Argentina. Arconada & Ramos (2002) investigated the pseudohermaphroditism of *Spathogyna fezi* (Altimira, 1960), a member of the Hydrobiidae family. The analysis of other freshwater gastropods has provided information on the gametogenesis of species belonging to the families Tateidae e.g., *Potamopyrgus antipodorum* (Gray, 1843), an invasive species from New Zealand that has colonized Australia, Europe, and the Americas (Tair-Abbaci & Garric, 2012), and Ampullariidae e.g., *Pomacea canaliculata* (Lamarck, 1801), *Pomacea scalaris* (D’Orbigny, 1835), and *Asolene platae* (Maton, 1809), species from Argentina (Martin, 1986; Martin, 1987; Martin, 1992). In México, Carreón-Palau et al. (2003) studied the reproductive system of *Pomacea padula catemacensis* (Baker, 1922); in Taiwan Wu et al. (2011) studied the reproduction of the invasive the South American apple snails, *Pomacea canaliculata* (Lamarck, 1822) and *Pomacea scalaris* (d’Orbigny, 1835) (Ampullariidae).

The work reported here was aimed at contributing essential information on the reproductive process and strategy of a species of the genus *Heleobia* through histological examination of the reproductive system of the gonochoric species *H. piscium*, along with a determination of the gonadal maturation stages, oogenesis, and spermatogenesis under natural conditions.

## Material and Methods

The specimens were collected in the Multiple-Use Natural Reserve Isla Martín García (authorized by the Dirección de Áreas Naturales Protegidas-OPDS Provincial Authority for Sustainable Development, Buenos Aires-La Plata Argentina. Authorization granted by note under docket number 2578-1530/2005-0), and deposited in the Malacological Collection at the Museo de La Plata, Buenos Aires Province, Argentina (MLP-Ma). Monthly collections were made from August 2005 through June 2007 in the eastern part of the island Arena Beach (34°11′09″S, 58°15′09″W) (Isla Martín García in located in the upper Rio de la Plata), to the south of the mouth of the Uruguay river. This coastal stretch is delimited by the Canal del Infierno, which is characterized by a remarkable exposure to strong winds from the southeast. This area constitutes an outcropping of the Brazilian massif of Precambrian crystalline basement rock upon which lie sediments of the Holocene and Pleistocene epochs of the Quaternary Period (Ravizza, 1984).

Samples of *H. piscium* were taken by hand in the coastal drainage channels formed by tidal erosion. A total 1,306 specimens were collected. The ratio of male to female specimens was 1 to 1.

In the histological analysis of gonads 10 individuals being females and 10 males from different sizes at random, for each climatic season (summer, autumn, winter and spring), for a total of 80 (eighty) were used. In the laboratory, the specimens were anesthetized with menthol crystals for 24 h before being fixed with Bouin or Raillet-Henry’s solution. After embedding the gonads in Paraplax, 10-µm cuts were made with a microtome and the resulting slices stained with hematoxylin-eosin (Gabe, 1968). Preparations of gonadal tissue at different stages of gametic development were observed under a Leica DM LS microscope and photomicrographs taken with a Tucsen USB2.0 camera connected to a microscope at 25× and 40× magnifications. After following the development of the gametogenic cycle in both sexes (from the gametogenic stages present in each individual) within an annual cycle throughout the two consecutive years of the study, the following stages were determined: early maturation, maturation, and evacuation.

## Results

*Heleobia piscium* is a dioecious oviparous species with internal fertilization. Male and female gonads form a spiral within the digestive gland and are composed of numerous follicles surrounded by connective tissue. The male gonad consists of a number of digitiform projections gathered in small groups. The tube that emerges from each of those groups is the *vas deferens* that travels from the base of the gonad to the anterior portion of the snail. Once in the ventral region, the *vas deferens* runs toward the stomach but, before reaching the posterior part of the stomach, turns back again toward the posterior part, finally returning to the stomach through the same route. In the posterior part of the stomach the *vas deferens* becomes rolled up, with the last section constituting the seminal vesicle. The male gonad is located in a small portion in the pallial cavity that is both the prostate gland.

The female reproductive system consists of the ovary, composed of follicles, the oviduct, the seminal receptacle, and the albumen gland. Like the penis, the gonadic follicles are dorsally located in the ventral or dorsolateral region of the digestive or hepatopancreatic gland, extending from the posterior portion of the stomach to the posterior end of the body. These follicles cover a smaller portion than that of the penis with respect to size of the digestive structure. The albumen gland is whitish and is topographically located in the posterior region of the pallial cavity.

### Histology

We conducted a histological study of the male and female gonads to ascertain the details the gonadogenesis development and the organization of the internal tissues. Therefore, to follow the evolution of the different stages of spermatogenesis and oogenesis, we mainly considered the characteristics and morphology of the sexual cells.

### Males

**Figure 1:**
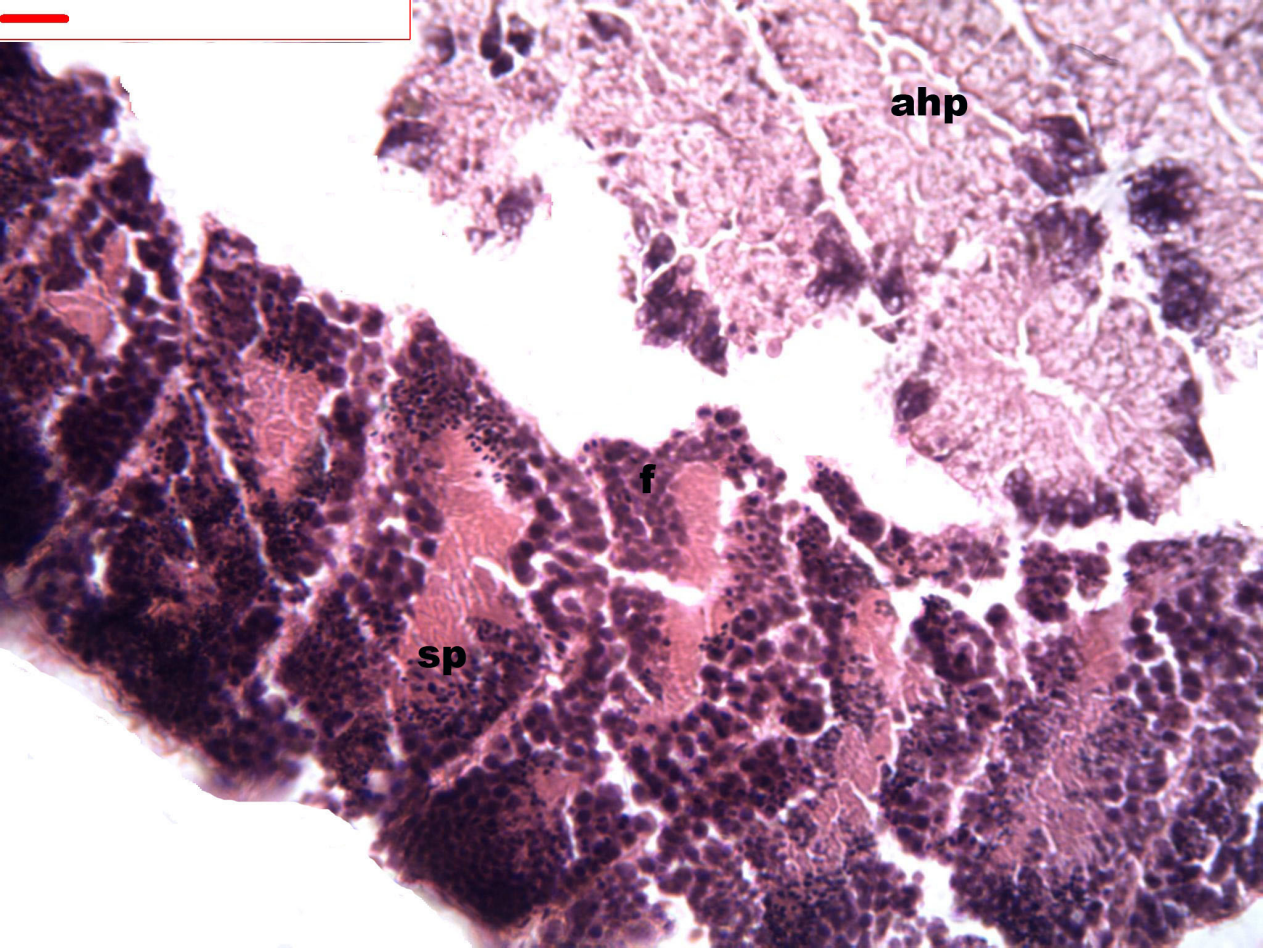
Stages of spermatogenesis. Maturation of the germinal sexual cells in the male acini. Spermatozoid, sp; follicle, f; hepatopancreatic acini, ahp.

**Figure 2:**
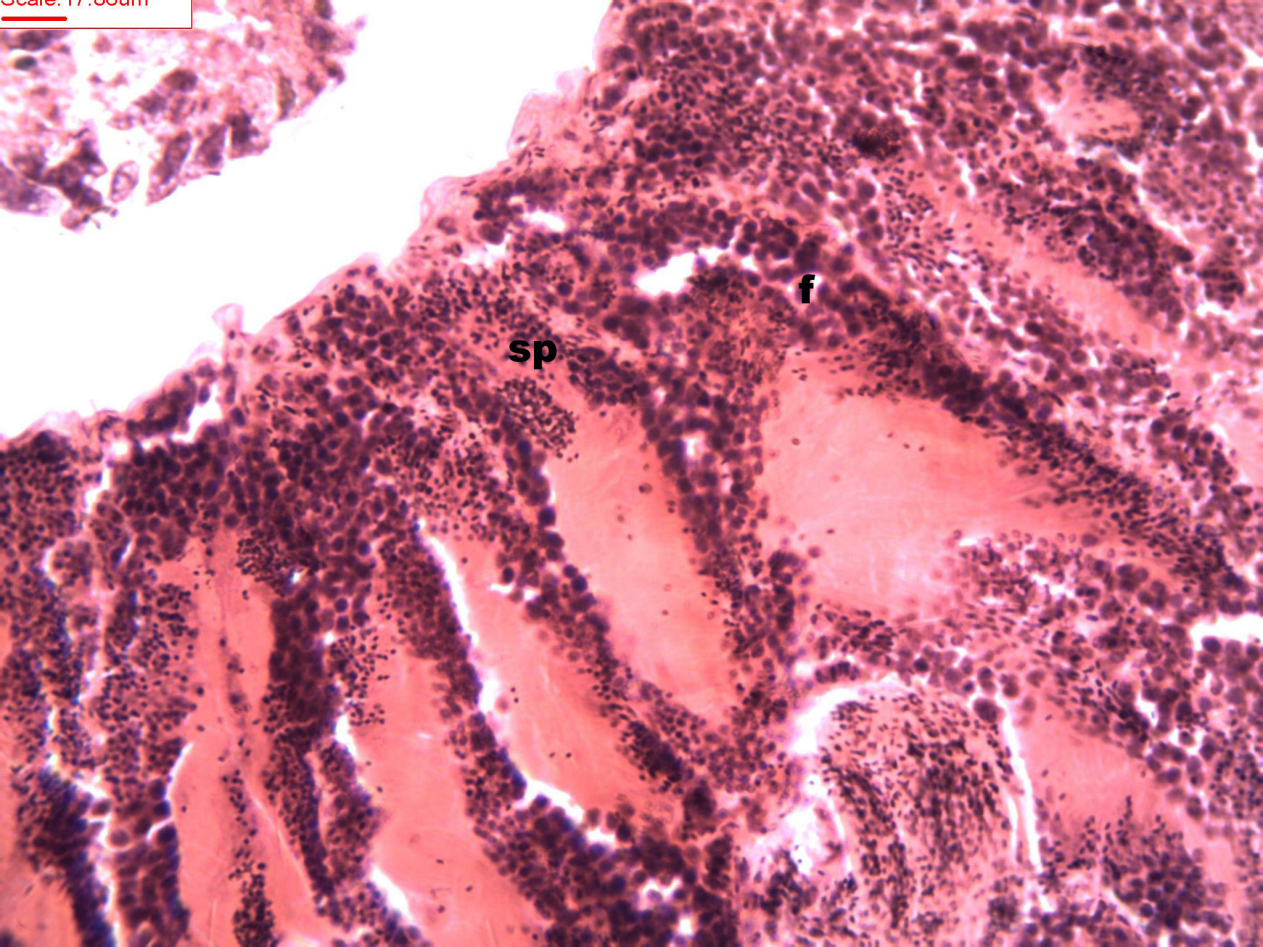
Stages of spermatogenesis. Maturation of the germinal sexual cells in the male acini. Spermatozoid, sp; follicle, f; hepatopancreatic acini, ahp.

**Figure 3:**
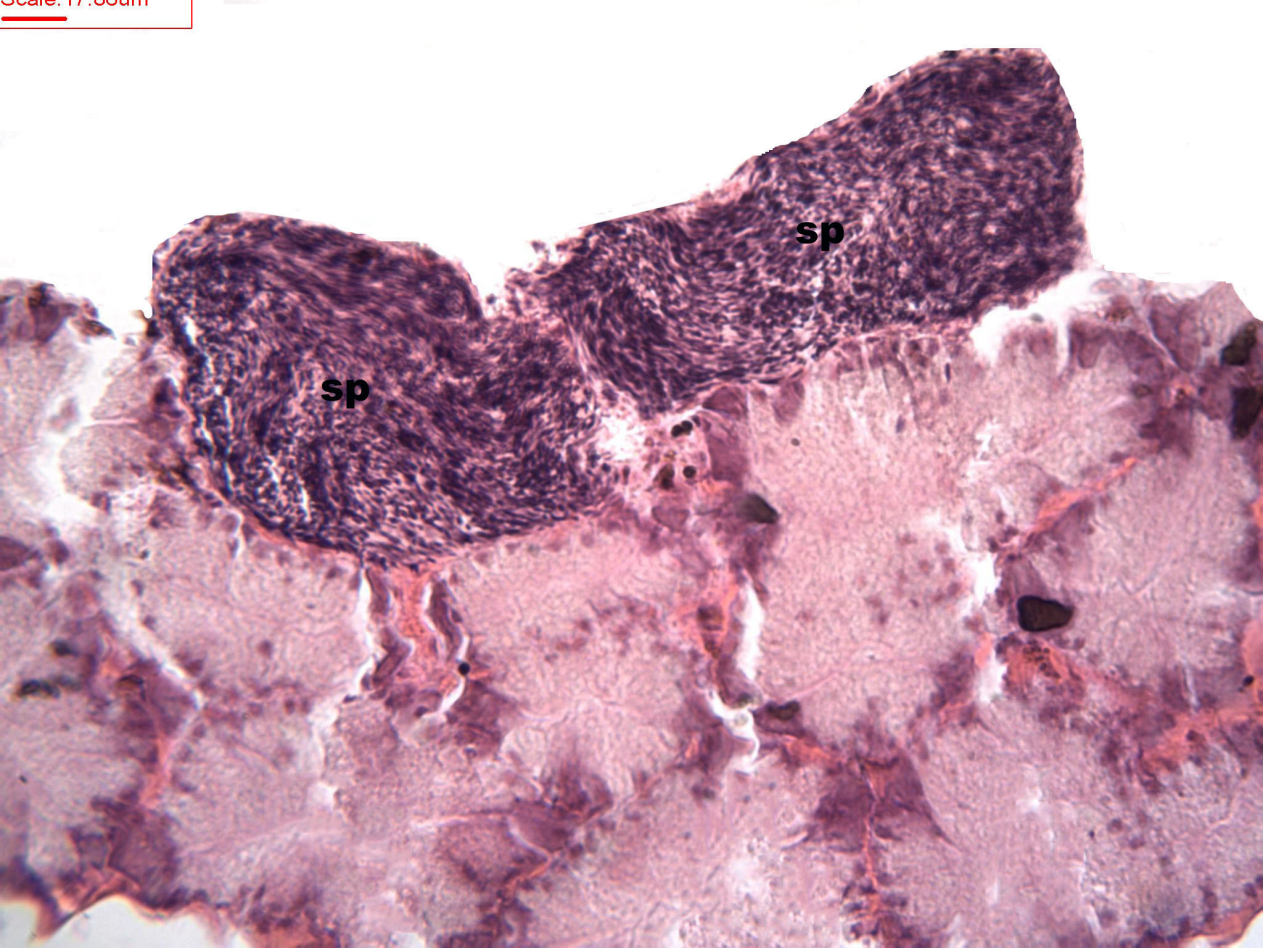
Stages of spermatogenesis. Maturation of the germinal sexual cells in the male acini. Spermatozoid, sp; follicle, f; hepatopancreatic acini, ahp.

During spermatogenesis, in the early maturation of the follicles large numbers of spermatogonia (3.64–4.55 µm) and spermatocytes (1.82–2.73 µm) are present. Upon complete maturation, spermatozoids become abundant in the lumen of the follicles, while the spermatocytes and spermatids (4.55 µm) are observed around the border of the acinar wall (Figs. 1, 2 and 3).

**Figure 4:**
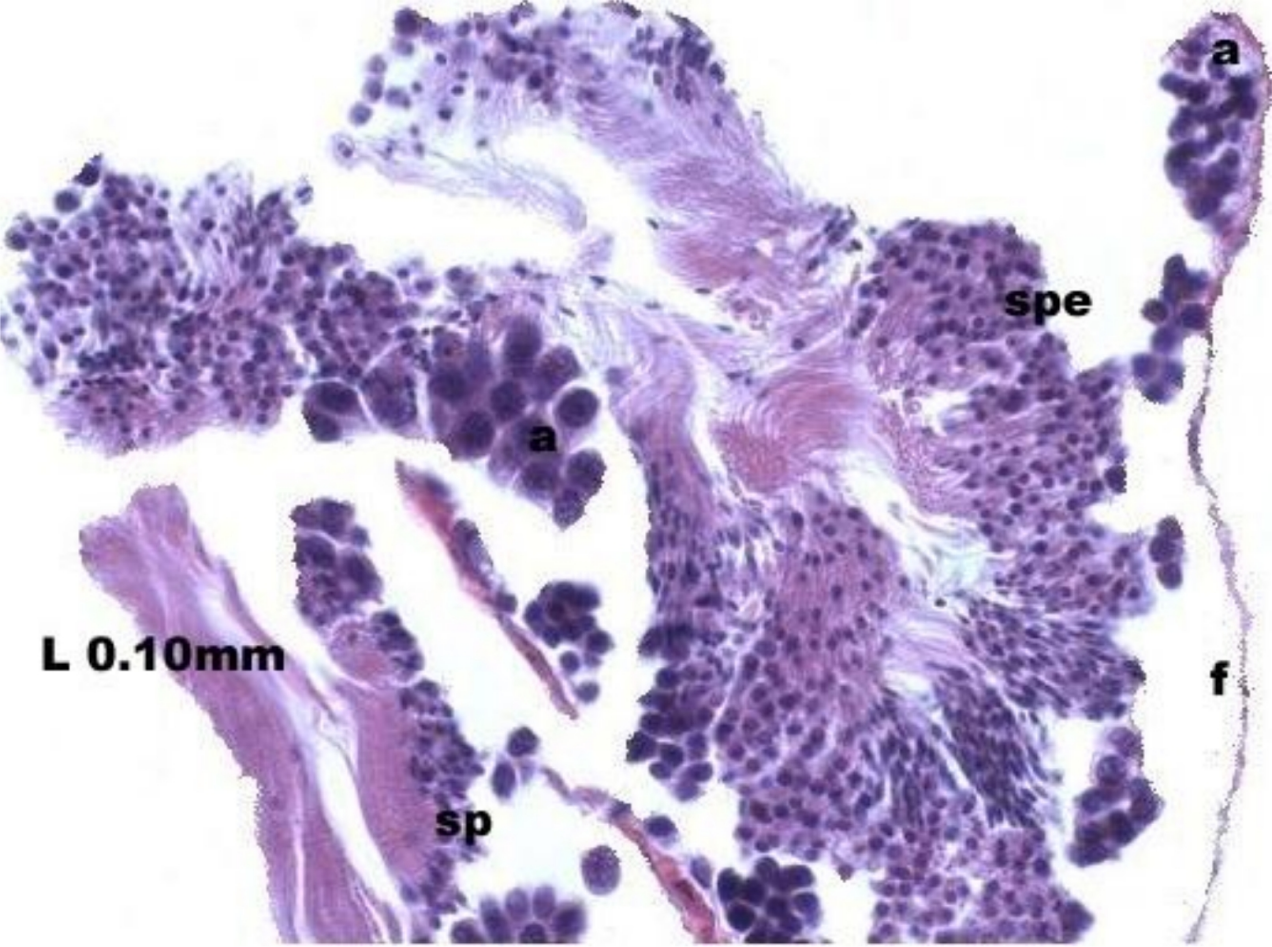
Stages of evacuation. The acinus characterized by the spermatozoids and spermatocytes, spe. Note the phagocytosis of the residual male cells by the amoebocytes, a spermatozoid, sp; follicle, f.

At the evacuation stage, the male follicles exhibit a different cytology, with the follicular lumen becoming almost empty and amoebocytes appearing that proceed to phagocytize the sexual cells remaining (Fig. 4). This process constitutes firm evidence that the evacuation of gametes has occurred. The histological cuts observed in the male revealed a tubular structure resembling tubes separated by connective tissue.

### Females

During oogenesis immature female gonadal follicles are present they measure less than 3 mm. Only developing oocytes were identified.

**Figure 5:**
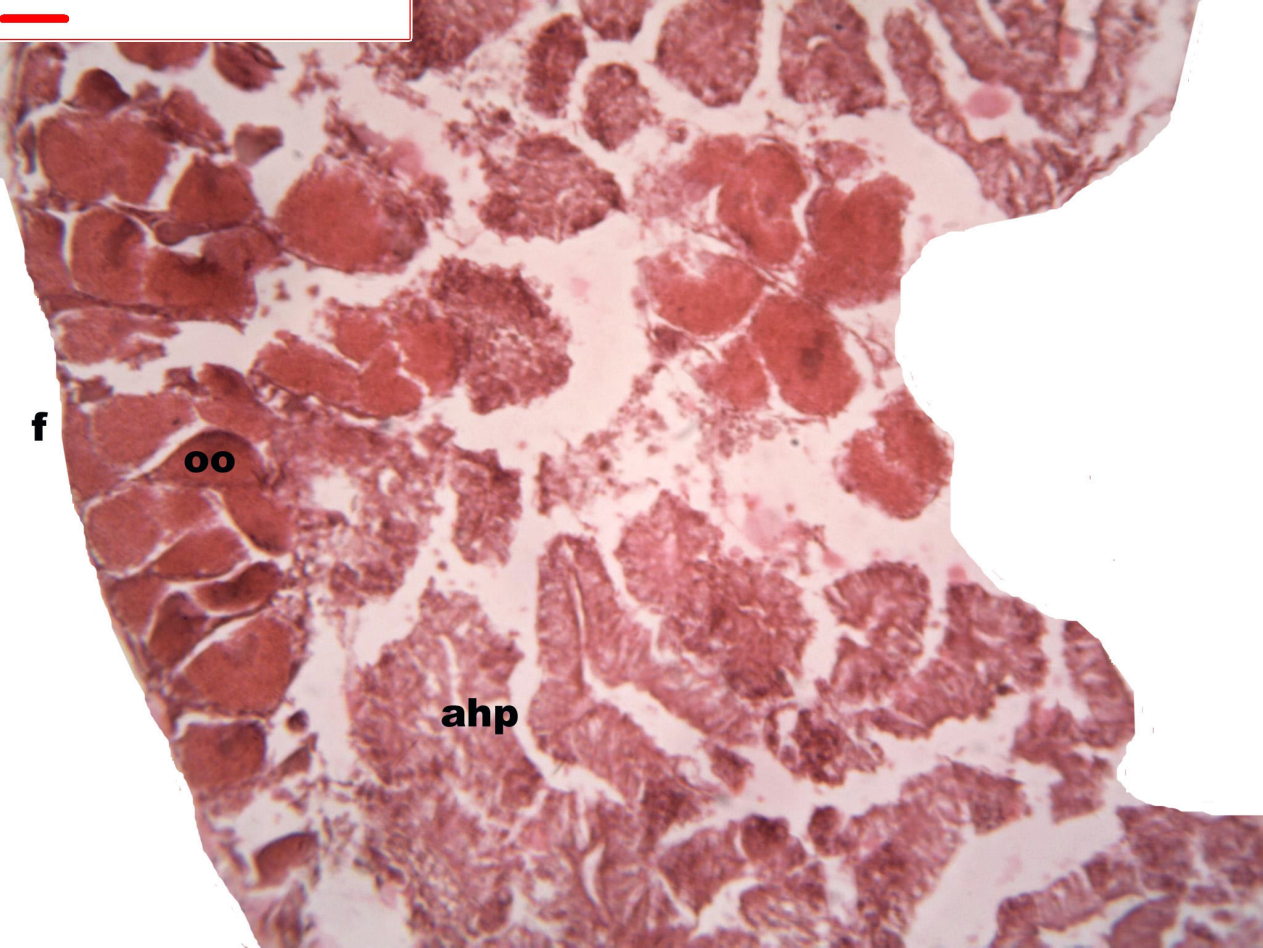
Stages of oogenesis. Yolk granules surrounded by oocytes. The nucleus was prominent during the stages of maturation. Oocyte, oo; follicle, f; hepatopancreatic acini, ahp.

**Figure 6:**
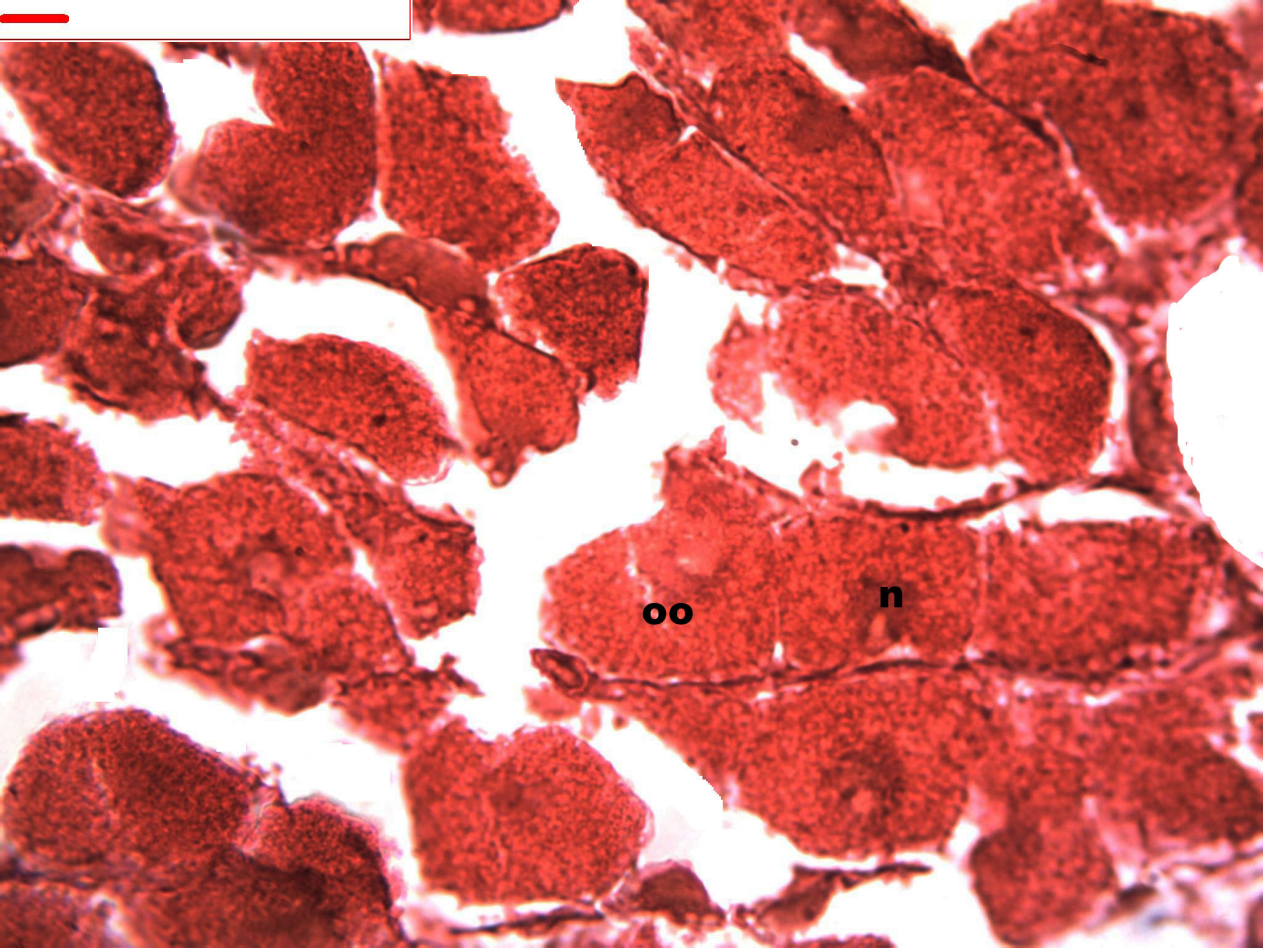
Stages of oogenesis. Stages of oogenesis. Detail of the nucleus and nucleolus was as prominent in the stages of maturation. Oocyte, oo; nucleo, n.

**Figure 7:**
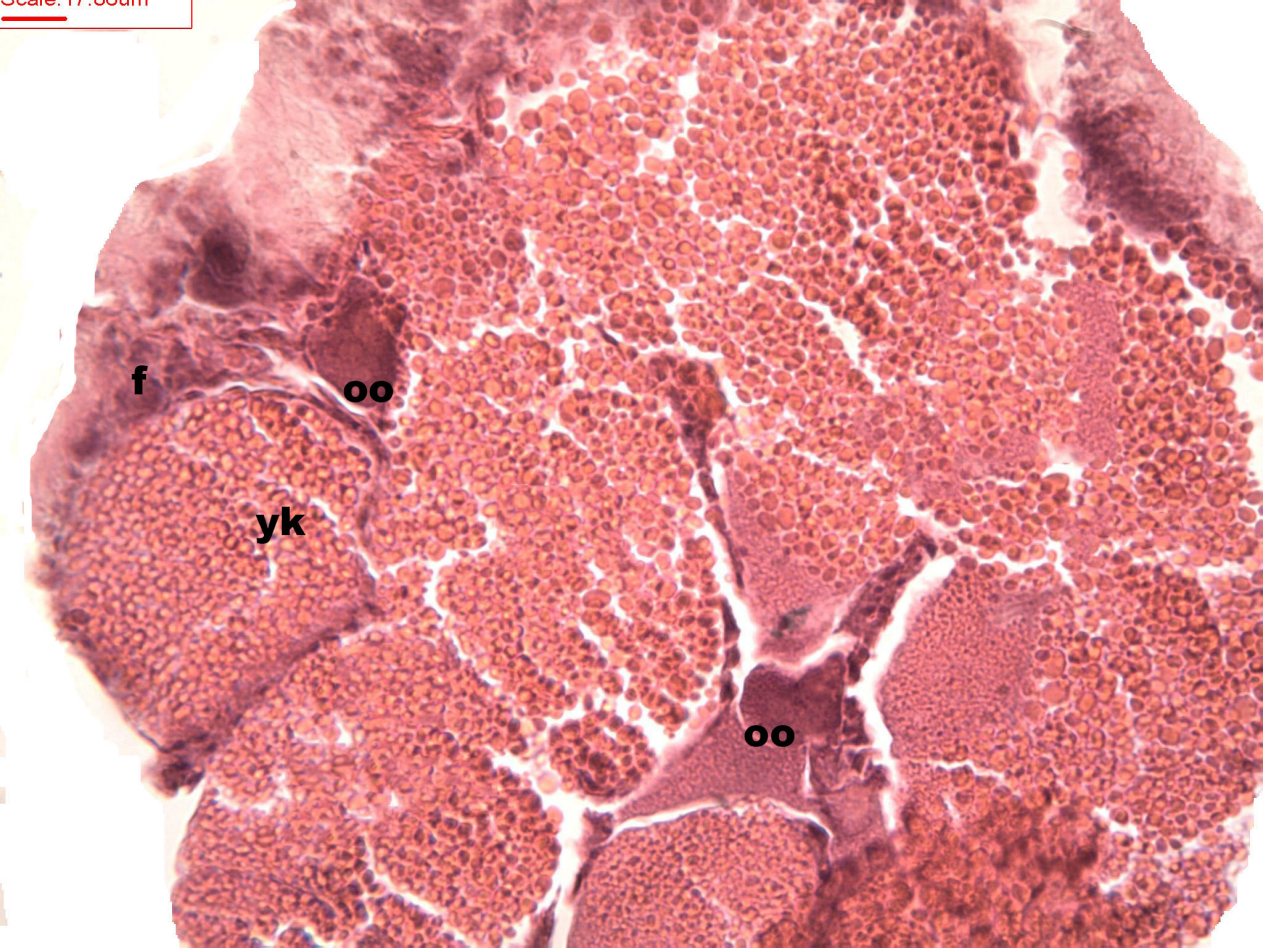
Stages of oosorption Stages of oosorption and degeneration of the oocytes and yolk granules. Oocyte, oo; yolk granules, yk; follicle, fc.

Follicles measuring less than 3 mm have only immature female gonads lacking vitellus granules along with developing oocytes. In female specimens that have reached a total length of 3–4 mm, the vitellogenic oocytes occupy these gonadic follicles. This stage, in fact, constitutes the indication that gonadal maturation has been completed (Figs. 5 and 6). Once reaching complete maturation, the female gonads begin evacuating their sexual cells. This evacuation is revealed by a spreading out of vitellus granules and a thinning of the oocytes, those being attached to the now extremely thin follicular walls (Fig. 7). At this stage cells referred to as amoebocytes with well colored nuclei located at one end of a wide, granulated cytoplasm appear to phagocytize residual oocytes. The histological cuts observed in females revealed that female follicles are surrounded by connective tissue. The small number of oocytes in the follicles attached to these thin follicular walls constitutes the evidence that sexual-cell evacuation has occurred.

## Discussion

The female gonad of *H. piscium* presents a ribbon-like distribution with few follicles. The size and intrafollicular location of the oocytes agree with reports by Jong-Brink, Boer & Joose (1983). In the present work, the female gametogenic cycle involved fully mature oocytes that were surrounded by abundant yolk granules, as observed in marine Caenogastropoda (Covarrubias & Romero, 2009). The presence of vitellus granules revealed a difference from other Caenogastropoda, such as the freshwater species of the Ampullariidae family that do not show such cells surrounding oocytes during the maturation stage (Martin, 1986; Martin, 1987; Martin, 1992).

The maturation stage for both males and females took place from January through October. During March, April, and May (the Southern-Hemisphere autumn) male and female individuals measuring 3.0–4.5 mm were at the early maturation stage. Complete maturation was observed during August, September, and October in specimens measuring 5.0–5.5 mm. Tair-Abbaci & Garric (2012) in the histological study and gonadal development of *Potamopyrgus antipodorum* showed early gonadogenesis when they reached 2 mm size, in *H. piscium* observed for about the same size.

The male gonad was found to be distributed in a centripetal form similar to that observed in other Caenogastropods of the Littoridinidae family (e.g., *Echinolittorina peruviana* (Lamarck, 1822); Castillo & Brown, 2008). This characteristic gives a variegated appearance to the gonad–digestive-gland complex, a formation quite different from a clearly separated gonad and digestive gland, or from digestion in a topographically separate location in other species. Hershler & Ponder (1998) described the same form of gonadal structure in several species of Hydrobiidae and based their description on the genital characteristics of *Spurwinkia salsa* (Pilsbry, 1905). Carreón-Palau et al. (2003) in *Pomacea patula catemacensis* (Baker 1922) and Wu et al. (2011) observed that the gonad of *Pomacea canaliculata* and *Pomacea scalaris* is in close contact with the digestive gland, which constitutes the visceral coil.

The evacuation of the gametes started in November and ended in February (the Southern-Hemisphere summer). Cazzaniga (1982) had observed in *L. parchappii*, among southern snail populations, that the maturation stage for both males and females took place from January to September (spring–summer) with the evacuation of the gametes starting in September and ending in January for each annual cycle of that species in Argentina. *Heleobia piscium* exhibits a reproductive cycle without any resting period, a characteristic shared with members of the genera *Pomacea* (Perry, 1811) and *Asolene* (D’Orbigny, 1837) studied by Martin (1986); Martin (1987); Martin (1992).

This present analysis of the population of *H. piscium* constitutes the first study on the gametogenic cycle of a species of Cochliopidae. Both the males and the females within the population displayed a synchronous gametogenic cycle. These results indicate that the *H. piscium* populations exhibit a continuous reproductive cycle in the absence of any resting period.

The present work, conducted at the Multiple-Use Natural Reserve Isla Martín García (Argentina), provides essential information on the annual reproductive cycle of *H. piscium.* This species could be used as “test taxon” in the assessment of the possible long-term negative effects of herbicides that are used in irrigation canals to control the growth of submerged aquatic vegetation. Environmental applications such as these were reported by Hershler & Davis (1980), who suggested that the hydrobiid family is one of the most fundamental groups of invertebrates used as a food source for other organisms in North America and Europe.

## Additional Information and Declarations

### Field Study Permissions

The following information was supplied relating to field study approvals (i.e., approving body and any reference numbers): Dirección de Áreas Naturales Protegidas-OPDS Provincial Authority for Sustainable Development, Buenos Aires-La Plata Argentina. Authorization granted under docket number 2578-1530/2005-0.

### Data Availability

The following information was supplied regarding data availability: The specimens are deposited at División Zoología Invertebrados-Sección Malacología-Museo de La Plata, Facultad Ciencias Naturales y Museo. Universidad Nacional de La Plata. DZI-MLP-UNLP Number MLP MA14179-14180-14181-14182.
